# Repeatability of methylation measures using a QIAseq targeted methyl panel and comparison with the Illumina HumanMethylation450 assay

**DOI:** 10.1186/s13104-021-05809-z

**Published:** 2021-10-24

**Authors:** Chenglong Yu, Pierre-Antoine Dugué, James G. Dowty, Fleur Hammet, JiHoon E. Joo, Ee Ming Wong, Mahnaz Hosseinpour, Graham G. Giles, John L. Hopper, Tu Nguyen-Dumont, Robert J. MacInnis, Melissa C. Southey

**Affiliations:** 1 Precision Medicine, School of Clinical Sciences at Monash Health, Monash University, Victoria, Australia; 2 Cancer Council Victoria, Cancer Epidemiology Division, Melbourne, Australia; 3 Centre for Epidemiology and Biostatistics, Melbourne School of Population and Global Health, The University of Melbourne, Parkville, VIC Australia; 4 Department of Clinical Pathology, The Melbourne Medical School, The University of Melbourne, Melbourne, VIC Australia

**Keywords:** DNA methylation, Whole blood, Formalin-fixed paraffin-embedded, Illumina Infinium methylation arrays, Targeted bisulphite sequencing, Intraclass correlation coefficient, Bland–Altman plot

## Abstract

**Objective:**

In previous studies using Illumina Infinium methylation arrays, we have identified DNA methylation marks associated with cancer predisposition and progression. In the present study, we have sought to find appropriate technology to both technically validate our data and expand our understanding of DNA methylation in these genomic regions. Here, we aimed to assess the repeatability of methylation measures made using QIAseq targeted methyl panel and to compare them with those obtained from the Illumina HumanMethylation450 (HM450K) assay. We included in the analysis high molecular weight DNA extracted from whole blood (WB) and DNA extracted from formalin-fixed paraffin-embedded tissues (FFPE).

**Results:**

The repeatability of QIAseq-methylation measures was assessed at 40 CpGs, using the Intraclass Correlation Coefficient (ICC). The mean ICCs and 95% confidence intervals (CI) were 0.72 (0.62–0.81), 0.59 (0.47–0.71) and 0.80 (0.73–0.88) for WB, FFPE and both sample types combined, respectively. For technical replicates measured using QIAseq and HM450K, the mean ICCs (95% CI) were 0.53 (0.39–0.68), 0.43 (0.31–0.56) and 0.70 (0.59–0.80), respectively. Bland–Altman plots indicated good agreement between QIAseq and HM450K measurements. These results demonstrate that the QIAseq targeted methyl panel produces reliable and reproducible methylation measurements across the 40 CpGs that were examined.

**Supplementary Information:**

The online version contains supplementary material available at 10.1186/s13104-021-05809-z.

## Introduction

Methylation arrays, including the Illumina Infinium HumanMethylation450 (HM450K) [1] and MethylationEPIC (EPIC) [2] arrays, have enabled genome-wide measurement of DNA methylation in a number of contexts [3–10]. The application of these arrays has included large discovery initiatives in studies with large sample sizes, which has been possible thanks to compatibility with clinical samples, relatively low DNA input and the cost efficiency of the array platforms [2, 11]. However, beyond the hypothesis generating initiatives, it is common for researchers to pursue findings on alternative platforms [12–16]. These platforms often need to enable a researcher-defined genomic target, for exploration of DNA methylation in specific genomic regions, not limited by CpGs included in the array designs. Such alternative platforms often also need to support an expanded sample size to adequately validate findings from initial array-based discovery-focused studies.

Here we used a custom QIAseq targeted methyl panel (Qiagen, Hilden, Germany) to measure DNA methylation in DNA samples extracted from whole blood (WB) and formalin-fixed paraffin-embedded (FFPE) tissues. The objectives of this study were to evaluate the repeatability of methylation measures by comparing the same technology duplicates (QIAseq) and different technology replicates (QIAseq and HM450K) based on the same DNA samples. The results will provide information for designing and conducting future larger studies aimed at targeted DNA methylation characterization.

## Main text

### Materials and methods

#### Sample selection

The DNA samples included in this study were selected from participants in the Australian Breast Cancer Family Registry [17, 18]. We included 26 DNA samples extracted from WB and 7 DNA samples extracted from FFPE breast cancer tissues. As shown in Table 1, 7 WB and 3 FFPE DNA samples were duplicated, i.e. duplicated DNA samples were included in the QIAseq targeted methyl panel sequencing, which we used to assess repeatability. Additionally, 25 of the 26 WB DNA samples and all 7 FFPE DNA samples had DNA methylation measured using the HM450K assay, as described previously [18–21], thus these samples were used to assess repeatability when using either of QIAseq or HM450K. All duplicated samples were from the same DNA extraction.

**Table 1:** Forty-one CpGs and their intraclass correlation coefficients (ICC) using 1) duplicated samples both measured using Qiagen targeted methyl panel, 2) replicated samples measured using Qiagen targeted methylated panel and Illumina HM450K assay

CpGs included on the QIAseq targeted methyl panel (and Illumina EPIC assay)			Repeatability (ICC, 95% CI), using duplicated samples both measured with Qiagen targeted methyl panel			Repeatability (ICC, 95% CI), using replicated samples measured with Qiagen targeted methyl panel and Illumina HM450K assay		
CpG^^^	Chr	Position^⁑^	WB samples 7 pairs	FFPE samples 3 pairs	Combined samples 10 pairs	WB samples 25 pairs	FFPE samples 7 pairs	Combined samples 32 pairs
cg18072778^*^	1	148,203,924	0.54 (0–0.90)	0.37 (0–0.94)	0.85 (0.54–0.96)	0 (0–0.39)	0.4 (0–0.88)	0.76 (0.56–0.88)
cg04546999^*^	1	152,956,430	0.19 (0–0.75)	0.80 (0–0.99)	0.92 (0.74–0.98)	0.09 (0–0.47)	0 (0–0.68)	0.8 (0.63–0.9)
cg17714793	1	153,538,431	0 (0–0.63)	0.99 (0.47–1)	0.72 (0.24–0.92)			
cg01608070	1	157,853,274	0.98 (0.89–1)	0.46 (0–0.96)	0.96 (0.84–0.99)			
cg21501207	1	162,383,000	0.89 (0.56–0.98)	0.28 (0–0.94)	0.80 (0.43–0.94)			
cg26237810	1	200,669,215	0.52 (0–0.89)	0.96 (0–1)	0.78 (0.29–0.95)			
cg26354017^*^	1	205,819,088	0.92 (0.64–0.98)	0.70 (0–0.97)	0.94 (0.78–0.98)	0.83 (0.67–0.92)	0.46 (0–0.86)	0.83 (0.69–0.92)
cg14159672^*^	1	205,819,179	0.95 (0.71–0.99)	0.96 (0.27–1)	0.98 (0.94–1.00)	0.79 (0.57–0.9)	0.76 (0.16–0.95)	0.82 (0.66–0.91)
cg14893161^*^	1	205,819,252	0.92 (0.63–0.98)	0.99 (0.59–1)	0.97 (0.89–0.99)	0.94 (0.86–0.97)	0.73 (0–0.94)	0.93 (0.86–0.96)
cg05841700	1	205,819,384	0.96 (0.80–0.99)	0.68 (0–0.98)	0.96 (0.88–0.99)			
cg24503407^*^	1	205,819,493	0.92 (0.65–0.98)	0 (0–0.96)	0.95 (0.82–0.99)	0.87 (0.74–0.94)	0.8 (0.02–0.98)	0.88 (0.76–0.94)
cg16334093	1	205,819,601	0.87 (0.43–0.97)	0.64 (0–0.97)	0.87 (0.54–0.97)			
cg07157834^*^	1	205,819,610	0.90 (0.47–0.98)	0 (0–0.89)	0.94 (0.75–0.98)	0.6 (0.29–0.81)	0.26 (0–0.82)	0.65 (0.39–0.81)
cg20004147	2	65,718,931	0.98 (0.92–1)	0.98 (0.47–1)	0.98 (0.94–1.00)			
cg21824770	2	243,012,164	0.28 (0–0.78)	0 (0–0.95)	0.88 (0.58–0.97)			
cg10123377	3	42,387,525	0.97 (0.87–0.99)	0.68 (0–0.97)	0.92 (0.72–0.98)			
cg01760119	3	101,661,383	0.12 (0–0.7)	0.87 (0–0.99)	0.98 (0.93–1.00)			
cg12012426^†^	4	1,366,464	–	–	–	–	–	–
cg19704288^*^	4	1,582,182	0.24 (0–0.79)	0.76 (0–0.98)	0.81 (0.43–0.95)	0 (0–0.38)	0.81 (0.33–0.96)	0.82 (0.66–0.91)
cg02722613^*, #^	4	25,162,899	0.75 (0.18–0.94)	0 (0–0.89)	0.77 (0.38–0.93)	0.87 (0.71–0.94)	–	–
cg19182683	4	183,730,519	0.98 (0.91–1)	0.94 (0–1)	0.97 (0.89–0.99)			
cg07158503^*, #^	5	135,415,693	0.63 (0–0.92)	0.96 (0.12–1)	0.79 (0.35–0.94)	0.55 (0.22–0.77)	–	–
cg11608150^*^	5	135,415,949	0.71 (0.02–0.93)	0.17 (0–0.97)	0.61 (0.03–0.89)	0.91 (0.81–0.96)	0.44 (0–0.94)	0.84 (0.7–0.92)
cg06478886^*^	5	135,416,030	0.79 (0.24–0.96)	0.98 (0.44–1)	0.85 (0.54–0.96)	0.94 (0.86–0.97)	0.31 (0–0.83)	0.9 (0.8–0.95)
cg04481923^*^	5	135,416,206	0.74 (0.05–0.95)	0 (0–0.89)	0.23 (0–0.71)	0.59 (0.28–0.78)	0.74 (0.04–0.95)	0.62 (0.34–0.79)
cg06536614^*^	5	135,416,381	0.81 (0.24–0.95)	0.47 (0–0.95)	0.63 (0.08–0.88)	0.71 (0.46–0.86)	0 (0–0.68)	0.7 (0.47–0.84)
cg25340688^*^	5	135,416,398	0.79 (0.21–0.96)	0.99 (0.66–1)	0.85 (0.47–0.95)	0.65 (0.36–0.83)	0.50 (0–0.88)	0.7 (0.47–0.84)
cg26896946^*^	5	135,416,405	0.82 (0.29–0.97)	0.55 (0–0.96)	0.76 (0.29–0.93)	0.68 (0.4–0.84)	0.31 (0–0.81)	0.72 (0.53–0.85)
cg00124993^*^	5	135,416,412	0.82 (0.21–0.96)	0.96 (0.13–1)	0.87 (0.55–0.97)	0.71 (0.45–0.86)	0.64 (0–0.92)	0.73 (0.52–0.86)
cg18797653^*^	5	135,416,613	0.89 (0.54–0.97)	0 (0–0.87)	0.89 (0.59–0.97)	0.50 (0.14–0.74)	0.76 (0.14–0.95)	0.56 (0.25–0.75)
cg09483595^*^	5	158,878,381	0.98 (0.91–1)	0.71 (0–0.98)	0.92 (0.68–0.98)	0 (0–0.4)	0.61 (0–0.91)	0.94 (0.88–0.97)
cg13373914	7	67,323,067	0.12 (0–0.74)	1.00 (0.94–1)	0.90 (0.63–0.98)			
cg05141217	8	28,491,379	0.96 (0.80–0.99)	1.00 (0.9–1)	0.97 (0.87–0.99)			
cg26708920	10	13,826,318	0.94 (0.72–0.99)	0.96 (0.01–1)	0.96 (0.86–0.99)			
cg20054939^*^	12	133,614,314	0.66 (0–0.92)	0 (0–0)	0.08 (0–0.63)	0 (0–0.41)	0 (0–0.72)	0 (0–0.36)
cg20124410	13	107,333,224	0.58 (0–0.9)	0 (0–0.87)	0.32 (0–0.73)			
cg10829391	14	101,069,717	0.95 (0.75–0.99)	0.59 (0–0.97)	0.87 (0.57–0.97)			
cg26748794^*^	16	88,804,052	0.97 (0.84–0.99)	0.59 (0–0.97)	0.89 (0.63–0.97)	0.67 (0.41–0.83)	0.69 (0.01–0.92)	0.67 (0.41–0.83)
cg20443278^*^	17	77,962,099	0.62 (0–0.91)	0.78 (0–0.99)	0.96 (0.85–0.99)	0 (0–0.38)	0.24 (0–0.8)	0.83 (0.67–0.91)
cg14150973^*^	19	40,950,432	0 (0–0.67)	0 (0–0.88)	0 (0–0.54)	0 (0–0.39)	0 (0–0.65)	0 (0–0.35)
cg17884856^*^	20	44,334,913	0.93 (0.69–0.99)	0.84 (0–0.99)	0.88 (0.59–0.97)	0.89 (0.79–0.95)	0.05 (0–0.68)	0.59 (0.32–0.78)

^^^CpG names used Illumina nomenclature

^*^CpGs included on the Infinium HM450K BeadChip. 24 and 22 CpGs were detected and passed QC on HM450K platform for WB and FFPE samples, respectively

^⁑^Genomic coordinates are based on human genome assembly hg19

^#^CpGs that did not pass QC on HM450K platform for the FFPE DNA samples

^†^CpGs that did not pass QC on the QIAseq targeted methyl panel

#### QIAseq targeted methyl panel library preparation and sequencing

The QIAseq targeted methyl panel was designed by the manufacturer (Qiagen, Hilden, Germany), to target 41 CpGs (in 28 genomic regions with total breadth of 8,673 bp, see Additional file 1: Table S1), identified in our previous studies of heritable methylation marks associated with cancer risk using the EPIC array [22]. Briefly, 100 ng WB and 200 ng FFPE DNA was bisulphite converted according to the manufacturer’s instructions with the EPITECT Fast Bisulfite Sequencing (BS) conversion kit. Bisulphite-converted DNA was used as the input template to create targeted libraries as per the QIAseq targeted methyl panel protocol. Enriched targeted sample libraries were amplified for 19 or 21 (genomic DNA or FFPE DNA) cycles respectively. Samples quantified by analysis on a D1000 HS ScreenTape (Agilent, Santa Clara, CA, USA) were pooled in equimolar concentrations and 10 pM pooled library was loaded for sequencing using 500 Cycle v2 MiSeq reagent kit (Illumina, San Diego, CA, USA).

Paired-end reads were mapped to the human genome reference (hg19) and methylation levels were called using the default settings of CLC Genomics Workbench (Qiagen). At a given CpG site, the DNA methylation level, a beta value between 0 (unmethylated) and 1 (fully methylated), was calculated as methylated coverage (the number of reads with evidence of methylation in this position) divided by context coverage (the number of reads conforming to the selected methylation context), see [23] for details. We assigned methylation values to “missing” when the context coverage was less than 30X.

#### Illumina Infinium HM450K array

HM450K BeadChip (Illumina, San Diego, CA, USA) DNA methylation measurements were available for 25/26 WB and 7/7 FFPE DNA samples assessed by QIAseq, and described previously [18–21]. Of the 41 CpGs of interest, which were previously identified as heritable cancer-associated marks using the EPIC array, only 24 were present on the HM450K array (Table 1) and therefore had available data for the tested samples. Two of these 24 CpGs (Illumina cg02722613 and cg07158503) did not pass quality control (QC) for the FFPE DNA samples (measures with a probe detection P-value of < 0.05 were assigned to “missing” and CpGs with > 20% missing values across samples were excluded in the original study [18]) and were excluded from further analysis (Table 1). Beta values obtained using the HM450K array data were used for comparison analysis with the QIAseq panel.

#### Data analysis

The distributions of paired beta values were represented graphically for repeated measures at each CpG, i.e. duplicated measures by the QIAseq panel, or replicated measures by QIAseq and HM450K platforms, which were assessed from the same DNA samples. We also used boxplots to show median and interquartile range (IQR) of beta values of QIAseq and HM450K platforms across different DNA sample types (WB, FFPE and sample types combined). Methylation values obtained for the two platforms were further compared using a Wilcoxon signed-rank test because the distributions of overall methylation values for the two platforms and methylation differences between the two platforms were not Gaussian (see “Results” section).

For each CpG, we estimated the repeatability of measurements by calculating the intraclass correlation coefficient (ICC) under a mixed effects model framework. The ICC is calculated as the variance within groups (i.e. duplicated DNA samples) means *V*_*G*_ divided by the sum of variances of the group-level and random error (residual) specific to each measure *V*_*R*_: *ICC* = *V*_*G*_/(*V*_*G*_ + *V*_*R*_). The ICC was estimated using the R package *rptR* [24] with a confidence interval (CI) quantified via parametric bootstrapping (1000 times). The ICC ranges from 0 to 1, and the higher the ICC, the higher the similarity between replicated values.

We also generated Bland–Altman plots to evaluate the agreement between repeated measures for each CpG, and used the 95% limits of agreement for each comparison (average difference ± 1.96 standard deviation (SD) of the difference). As the sample size for FFPE was small, we only examined Bland–Altman plots for WB and combined (WB and FFPE) DNA samples.

### Results

Average coverage of the targeted regions (Additional file 1: Table S1) sequenced using the QIAseq targeted methyl panel for the 26 WB, 7 FFPE and 33 samples combined was 447× (range 190×–1268×), 258× (range 66×–539×) and 408X (range 66×–1268×) with 92.4%, 90.4% and 92.0% reads mapped, respectively. Average context coverage of each of the 41 CpGs for WB, FFPE and combined DNA samples was also shown in Additional file 1: Table S2. One CpG at chr4:1,366,464 (Illumina cg12012426) was excluded from the analysis due to poor coverage (> 20% samples had less than 30× coverage), the remaining 40 CpGs across 15 chromosomes were retained for the analysis (Table 1).

The distributions of paired beta values for duplicated samples measured using the QIAseq panel (40 CpGs) and replicated samples measured using the QIAseq and HM450K platforms (24 CpGs) are shown in Additional file 1: Figures S1 and S2, respectively, showing good consistency of repeated measures.

**Fig. 1:**
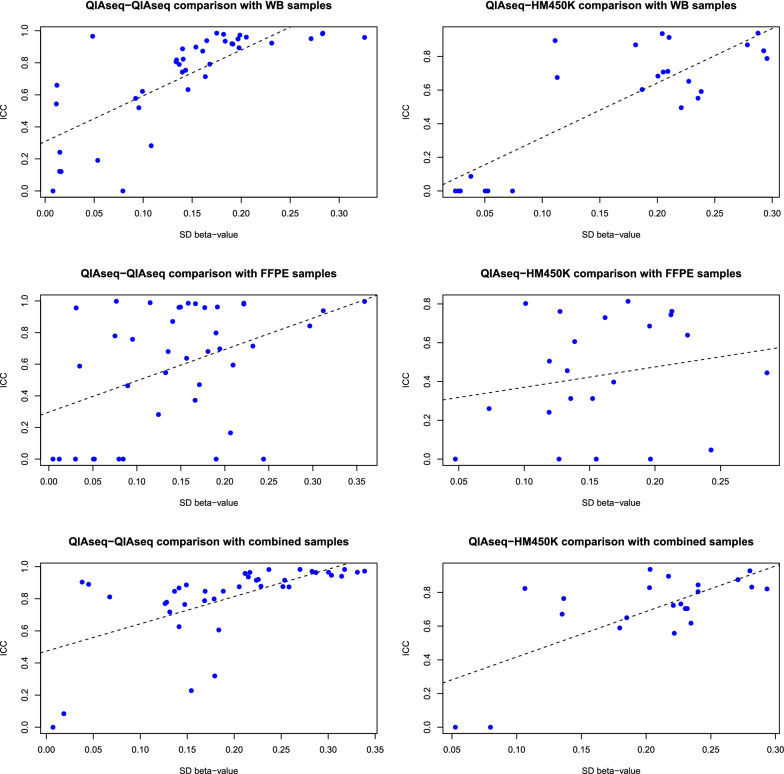
Relationship between CpG variability and intraclass correlation coefficient

Table 1 shows the repeatability (ICC) of the QIAseq panel measurements for all 40 CpGs. The mean (95% CI) ICC across 40 CpGs was 0.72 (0.62–0.81) for WB samples, 0.59 (0.47–0.71) for FFPE samples, and 0.80 (0.73–0.88) for the two sample types combined. The majority of CpGs, 33/40 and 36/40, had an ICC greater than 0.5 for WB and all sample types combined, respectively. The estimated ICC was low for CpGs with more extreme values (closer to 0 or 1), for which there was low methylation variability. For instance, Illumina cg20054939 and cg14150973 had poor repeatability (ICC < 0.1 in all samples combined) but the beta value SDs were lower than 0.02, as shown in Fig. 1 and Additional file 1: Figure S1.

Table 1 also shows the repeatability of methylation measures at 24 CpGs when using the QIAseq and HM450K technologies. The mean (95% CI) ICC across these 24 CpGs was 0.53 (0.39–0.68) for WB samples, 0.43 (0.31–0.56) for FFPE samples and 0.70 (0.59–0.80) when sample types were combined. Similar to observations made above, the majority of CpGs, 17/24 and 20/22, had an ICC greater than 0.5 for WB and all sample types combined, respectively, and those CpGs that had low ICC predominantly showed little variability (Fig. 1 and Additional file 1: Figure S2).

**Fig. 2:**
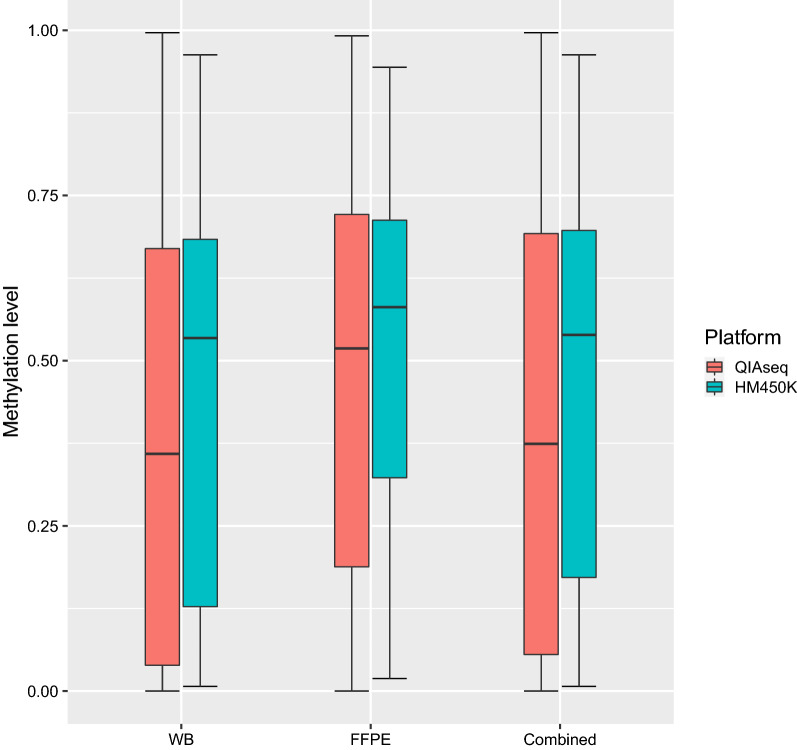
Overall methylation measures (24 CpGs) using Qiaseq targeted methyl panel or Illumina HM450K assay

Additional file 1: Figure S3 presents boxplots of beta values at each of the 24 CpGs for QIAseq and HM450K technology across sample types. We also show in Fig. 2 boxplots of methylation value distributions for all CpGs combined. Additional file 1: Figure S4 shows that the distributions of overall methylation values for the two platforms and methylation differences between the two platforms. As the distributions of methylation differences between the two platforms were not Gaussian, a Wilcoxon signed-rank test was used to compare them. On average, the QIAseq targeted panel produced lower methylation levels than those obtained using the HM450K assay; WB: median [IQR] for QIAseq: 0.36 [0.04–0.67] and HM450K: 0.53 [0.13–0.68], Wilcoxon signed-rank test, P < 2 × 10^–16^; FFPE: median [IQR] for QIAseq: 0.52 [0.19–0.72] and for HM450K: 0.58 [0.32–0.71], P = 0.01; all samples combined: median [IQR] for QIAseq: 0.37 [0.06–0.69] and HM450K: 0.54 [0.17–0.70], P < 2 × 10^–16^

The agreement of repeated measures using the QIAseq and HM450K platforms (24 CpGs) was also assessed using Bland–Altman plots as shown in Additional file 1: Figures S5 (WB) and S6 (sample types pooled), respectively. There was overall good agreement of the QIAseq and HM450K measures. For WB DNA, the mean of differences of the two measures was within the 95% limits of agreement at 21 of the 24 CpGs, all 25 samples were within the 95% limits of agreement at 10 of the 21 CpGs, and a maximum of 2 samples were beyond at the remaining 11 CpGs (Additional file 1: Figure S5). When sample types (WB and FFPE) were combined, the mean of differences of the two measures was within the 95% limits of agreement at all the 22 CpGs, all 32 samples were within the 95% limits of agreement at 4 of the 22 CpGs, and a maximum of 4 samples were beyond at the remaining 18 CpGs (Additional file 1: Figure S6). The figures showed that most outliers in the combined data were FFPE DNAs.

### Discussion

Overall, our results demonstrate that the QIAseq targeted methyl panel sequencing can produce reliable and repeatable methylation measures. Our analyses included ICCs and Bland–Altman plots, both showing that the QIAseq and HM450K platforms have overall good repeatability and agreement, especially for high-molecular weight WB DNA samples; this was despite the somewhat higher methylation values produced using the HM450K assay, particularly at lower ranges. The low ICCs observed at some CpGs, e.g. 7/40 had an ICC less than 0.5 for duplicated WB samples of QIAseq measurements, were essentially due to the low methylation variability observed at these CpGs (typically an SD across samples lower than 5% methylation), making it difficult to distinguish measures between samples, rather than poor measurement quality. Several factors may explain small differences in methylation levels between duplicates, including biological (e.g. average across a collection of cells) and technical (e.g. batch effects), so that the observed repeatability is necessarily imperfect, both within and across measurement technology.

QIAseq targeted methyl panel sequencing can not only reliably reproduce the Infinium array data but also enable the characterisation of DNA methylation in the surrounding genomic region. Although not presented here, the QIAseq technology provides information about the DNA methylation status of the 28 targeted regions, facilitating future extended analyses of the regions around the CpGs that we have identified to be associated with cancer risk—beyond the capacity of the genome-wide array technology. This platform can therefore support an expanded sample size to adequately validate findings from initial array-based discovery-focused studies and support further genomic region-focused exploration of DNA methylation.

## Limitations

Our study has three main limitations. First, only a limited number of CpGs were included in this analysis. These CpGs were of particular interest to us and may not be representative of all CpGs across the genome, however the included CpGs were spread across several chromosomes and genomic regions. Second, our sample sizes were small, especially for FFPE DNA samples and thus our findings would require further confirmation using more duplicate pairs. Nevertheless, our aim was not to obtain very precise estimation of ICCs but rather to confirm that these were good for most CpGs. Third, there was insufficient coverage (< 30×) for some targeted CpGs in some samples, which could be addressed by increasing sequencing depth.

## Supplementary Information

**Additional file 1:** Supplementary Tables and Figures.

## Data Availability

The datasets used and/or analysed during the current study are available from the corresponding author on reasonable request.

## Abbreviations

CpG: 5'-C-phosphate-G-3'
WB: Whole blood
FFPE: Formalin-fixed paraffin-embedded tissue
ICC: Intraclass correlation coefficient
HM450K: HumanMethylation450
EPIC: MethylationEPIC
BS: Bisulfite sequencing
QC: Quality control
IQR: Interquartile range
CI: Confidence interval
SD: Standard deviation
