# Supplementary material for: Repeatability of methylation measures using a QIAseq targeted methyl panel and comparison with the Illumina HumanMethylation450 assay
Source: BMC Res Notes. 2021 Oct 24;14:394. doi: 10.1186/s13104-021-05809-z (PMC8543877; doi:10.1186/s13104-021-05809-z)

Repeatability of methylation measures using a QIAseq targeted methyl panel and comparison with the Illumina HumanMethylation450 assay

Supplementary Information

This supplementary file includes 2 tables and 6 figures:

Table S1. Twenty-eight genomic regions in the QIAseq targeted methyl panel for this study.

Table S2. Average context coverage of each of the 41 CpGs for WB, FFPE and combined samples.

Figure S1. Distributions of paired beta values at 40 CpGs for duplicated samples measured using the QIAseq panel. Red and blue triangles: QIAseq.

Figure S2. Distributions of paired beta values at 24 CpGs for duplicated samples measured using QIAseq and HM450K platforms. Red triangle: QIAseq. Blue square: HM450K.

Figure S3. Boxplots with median and IQR of beta values at 24 CpGs for QIAseq and HM450K platforms across sample types.

Figure S4. Distributions of overall methylation values for QIAseq targeted methyl panel and Illumina HM450K assay and methylation differences between the two platforms (24 CpGs).

Figure S5. Bland-Altman plots of repeated measures for QIAseq and HM450K platforms (24 CpGs) for 25 WB samples. Solid line: mean of differences; black dashed line: mean of differences ± 1.96 standard deviation of the differences; red dashed line: difference = 0.

Figure S6. Bland-Altman plots of repeated measures for QIAseq and HM450K platforms (24 CpGs) for 32 combined samples (25 WB and 7 FFPE). Blue circle: WB. Red circle: FFPE. Solid line: mean of differences; black dashed line: mean of differences ± 1.96 standard deviation of the differences; red dashed line: difference = 0.

Table S1. Twenty-eight genomic regions covering 41 CpGs in the QIAseq targeted methyl panel for this study.

Chr.	Genomic region*	Length (bp)	CpGs covered^	Nearby gene
1	148203793 - 148204006	214	cg18072778	*PPIAL4F*
1	152956233 - 152956603	371	cg04546999	*SPRR1A*
1	153538249 - 153538582	334	cg17714793	*S100A2*
1	157853156 - 157853365	210	cg01608070	
1	162382834 - 162383043	210	cg21501207	*SH2D1B*
1	200669056 - 200669342	287	cg26237810	
1	205818927 - 205819749	823	cg26354017<br>cg14159672<br>cg14893161<br>cg05841700<br>cg24503407<br>cg16334093<br>cg07157834	*PM20D1*
2	65718838 - 65719047	210	cg20004147	
2	243012109 - 243012318	210	cg21824770	*LINC01237*
3	42387332 - 42387667	336	cg10123377	
3	101661366 - 101661574	209	cg01760119	*LOC152225*
4	1366368 - 1366589	222	cg12012426	*KIAA1530*
4	1582122 - 1582330	209	cg19704288	
4	25162696 - 25163099	404	cg02722613	*SEPSECS*
4	183730401 - 183730643	243	cg19182683	
5	135415589 - 135415797	209	cg07158503	*VTRNA2-1*
5	135415820 - 135416793	974	cg11608150<br>cg06478886<br>cg04481923<br>cg06536614<br>cg25340688<br>cg26896946<br>cg00124993<br>cg18797653	*VTRNA2-1*
5	158878240 - 158878522	283	cg09483595	*LOC285627*
7	67322939 - 67323147	209	cg13373914	
8	28491307 - 28491523	217	cg05141217	
10	13826111 - 13826503	393	cg26708920	*FRMD4A*
12	133614243 - 133614460	218	cg20054939	*ZNF84*
13	107333119 - 107333334	216	cg20124410	
14	101069509 - 101069841	333	cg10829391	
16	88803919 - 88804175	257	cg26748794	*FAM38A*
17	77961990 - 77962238	249	cg20443278	*TBC1D16*
19	40950319 - 40950623	305	cg14150973	*SERTAD3*
20	44334761 - 44335078	318	cg17884856	*WFDC10B*
		Total: 8,673		

*Genomic coordinates are based on human genome assembly hg19.
^CpG names used Illumina nomenclature.

Table S2. Average context coverage of each of the 41 CpGs for WB, FFPE and combined samples.

Illumina CpG name	Chr.	Position*	Average context coverage (X)		
			WB samples (N=26)	FFPE samples (N=7)	Combined samples (N=33)
cg18072778	1	148203924	447.1	156.9	385.5
cg04546999	1	152956430	309.0	278.1	302.5
cg17714793	1	153538431	384.7	516.0	412.5
cg01608070	1	157853274	434.0	191.4	382.5
cg21501207	1	162383000	378.4	266.7	354.7
cg26237810	1	200669215	262.5	90.0	225.9
cg26354017	1	205819088	472.4	417.9	460.8
cg14159672	1	205819179	502.8	604.7	524.5
cg14893161	1	205819252	576.7	605.6	582.8
cg05841700	1	205819384	751.3	626.3	724.8
cg24503407	1	205819493	350.1	156.6	309.1
cg16334093	1	205819601	435.7	306.1	408.2
cg07157834	1	205819610	424.4	277.4	393.2
cg20004147	2	65718931	296.9	57.9	246.2
cg21824770	2	243012164	248.7	112.0	219.7
cg10123377	3	42387525	505.5	250.7	451.5
cg01760119	3	101661383	368.1	173.1	326.7
cg12012426^	4	1366464	118.7	28.0	99.5
cg19704288	4	1582182	158.9	278.9	184.4
cg02722613	4	25162899	295.3	175.9	270.0
cg19182683	4	183730519	336.7	88.1	284.0
cg07158503	5	135415693	542.5	205.6	471.0
cg11608150	5	135415949	214.7	67.1	183.4
cg06478886	5	135416030	549.7	514.1	542.2
cg04481923	5	135416206	366.7	113.1	312.9
cg06536614	5	135416381	364.4	200.6	329.6
cg25340688	5	135416398	409.4	267.1	379.2
cg26896946	5	135416405	425.8	279.3	394.8
cg00124993	5	135416412	444.4	305.6	415.0
cg18797653	5	135416613	312.0	229.1	294.4
cg09483595	5	158878381	730.8	298.7	639.2
cg13373914	7	67323067	387.1	77.7	321.5
cg05141217	8	28491379	234.5	63.6	198.3
cg26708920	10	13826318	507.0	468.1	498.7
cg20054939	12	133614314	168.3	81.3	149.9
cg20124410	13	107333224	469.1	89.1	388.5
cg10829391	14	101069717	465.2	397.3	450.8
cg26748794	16	88804052	249.8	254.1	250.7
cg20443278	17	77962099	479.0	484.4	480.1
cg14150973	19	40950432	453.9	1266.0	626.2
cg17884856	20	44334913	543.6	180.1	466.5

*Genomic coordinates are based on human genome assembly hg19.
^CpGs that did not pass QC on the QIAseq targeted methyl panel.

Figure S1

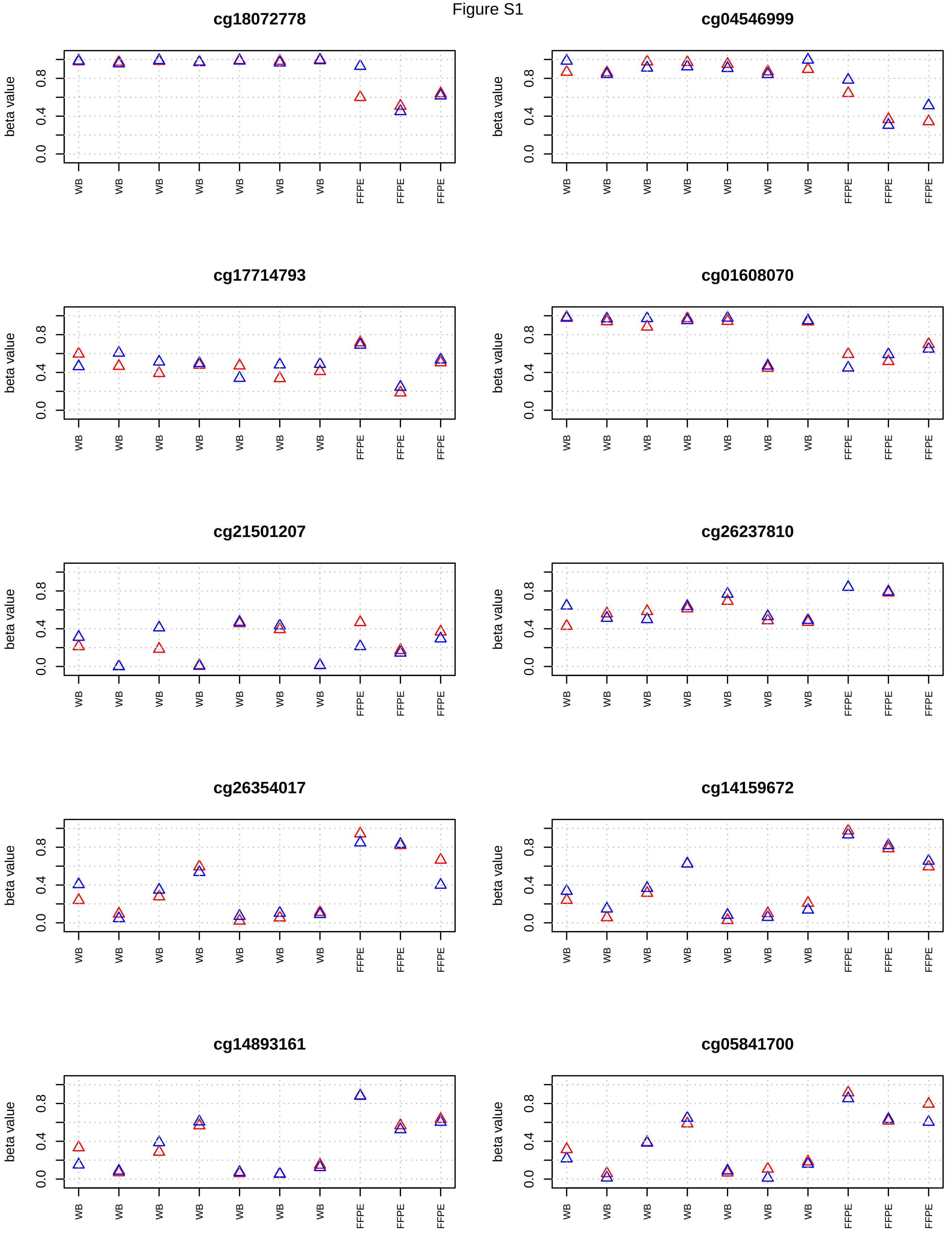
cg18072778
cg04546999
cg17714793
cg01608070
cg21501207
cg26237810
cg26354017
cg14159672
cg14893161
cg05841700
beta value
0.0
0.4
0.8
WB
FFPE

Figure S1 (extended)

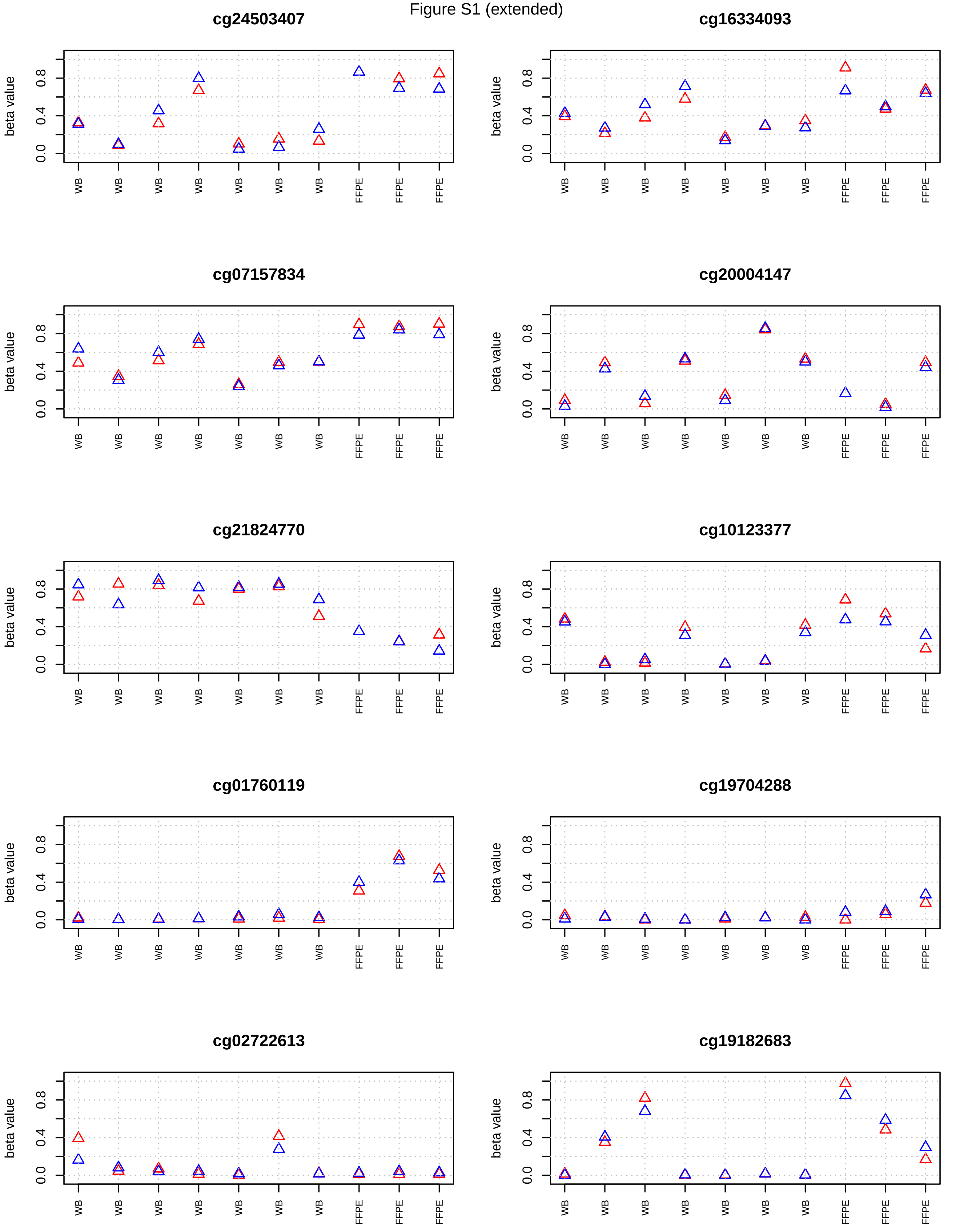

Figure S1 (extended)

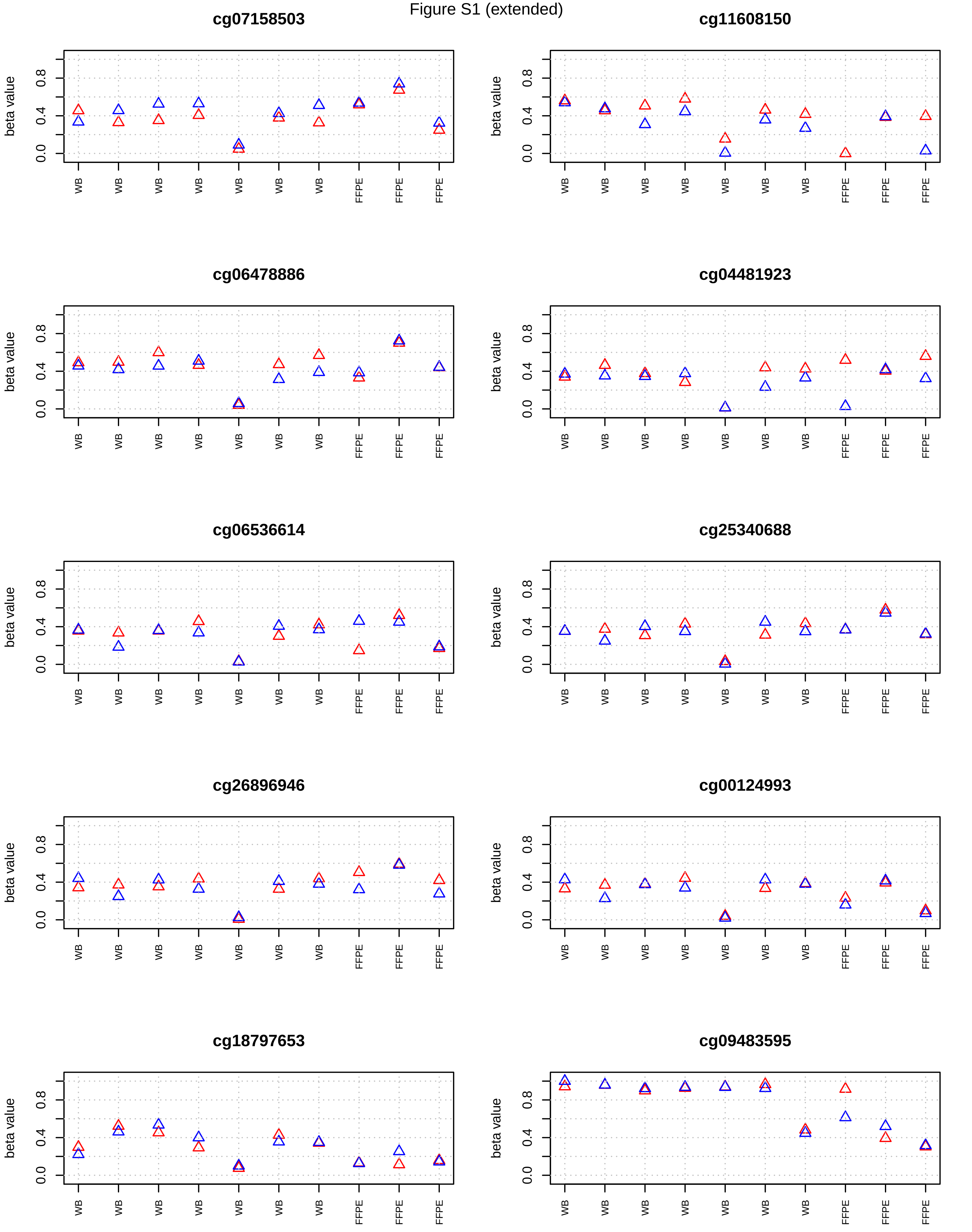

Figure S1 (extended)

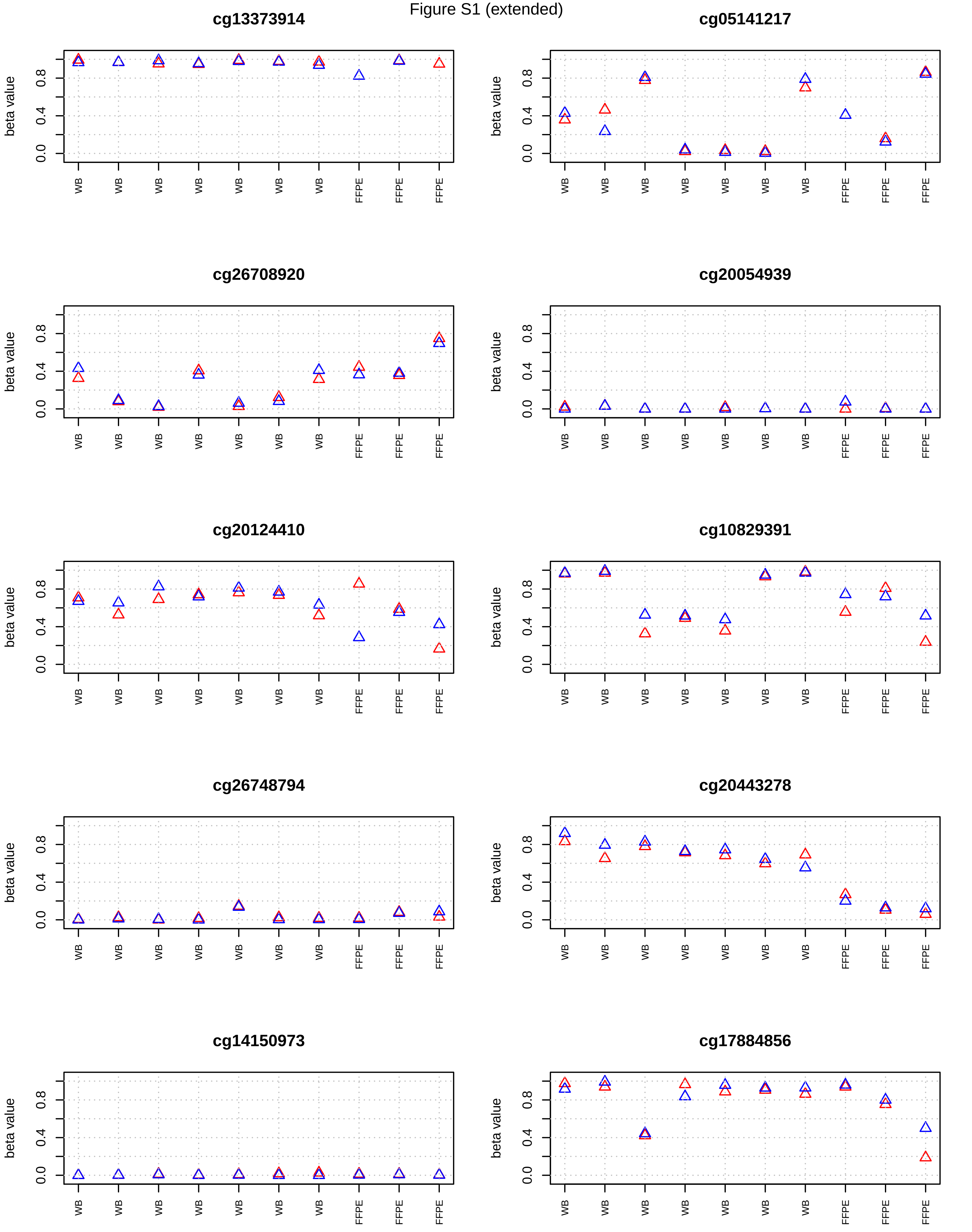

Figure S2

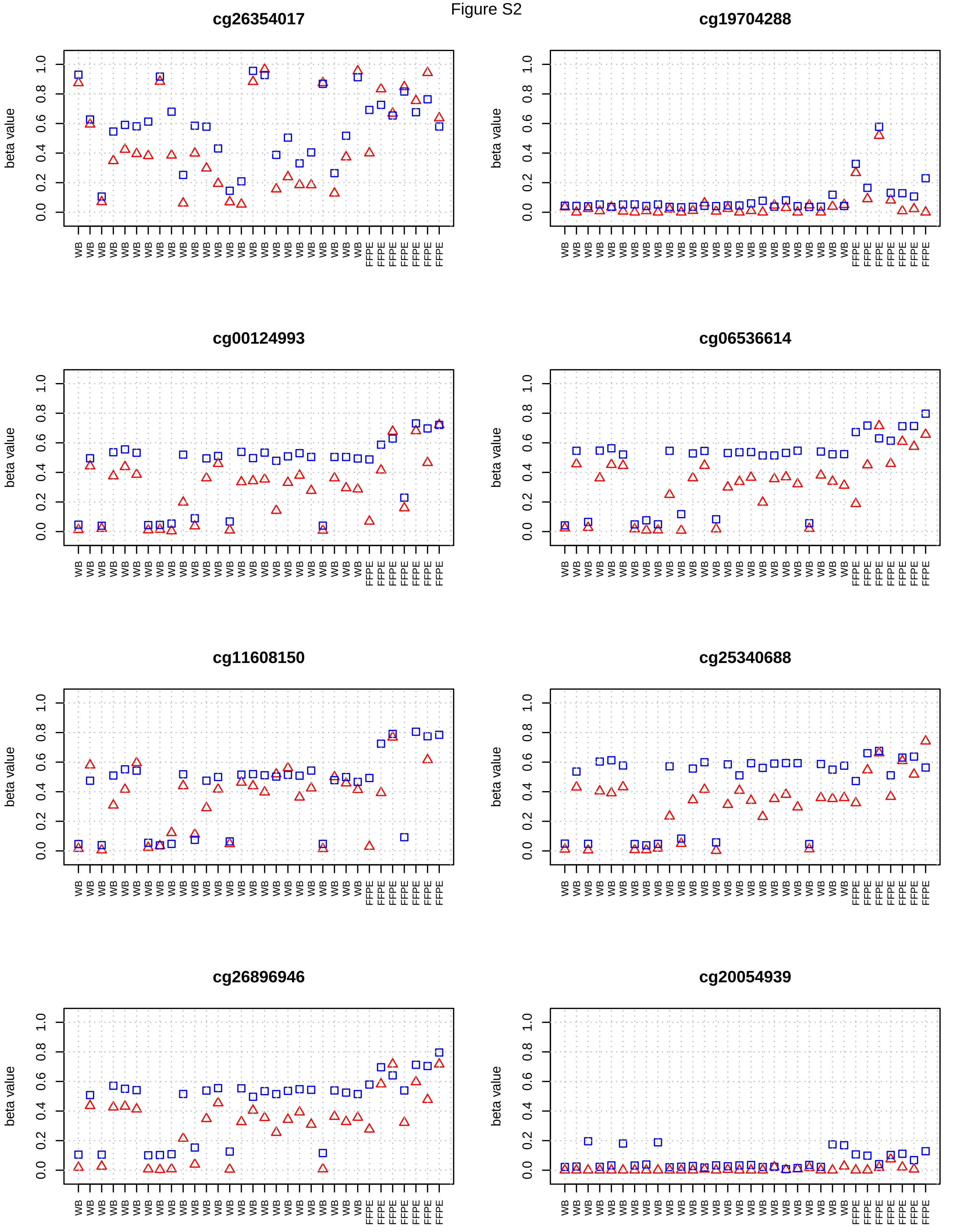
cg26354017
cg19704288
cg00124993
cg06536614
cg11608150
cg25340688
cg26896946
cg20054939
beta value
0.0 0.2 0.4 0.6 0.8 1.0
WB
FFPE

Figure S2 (extended)

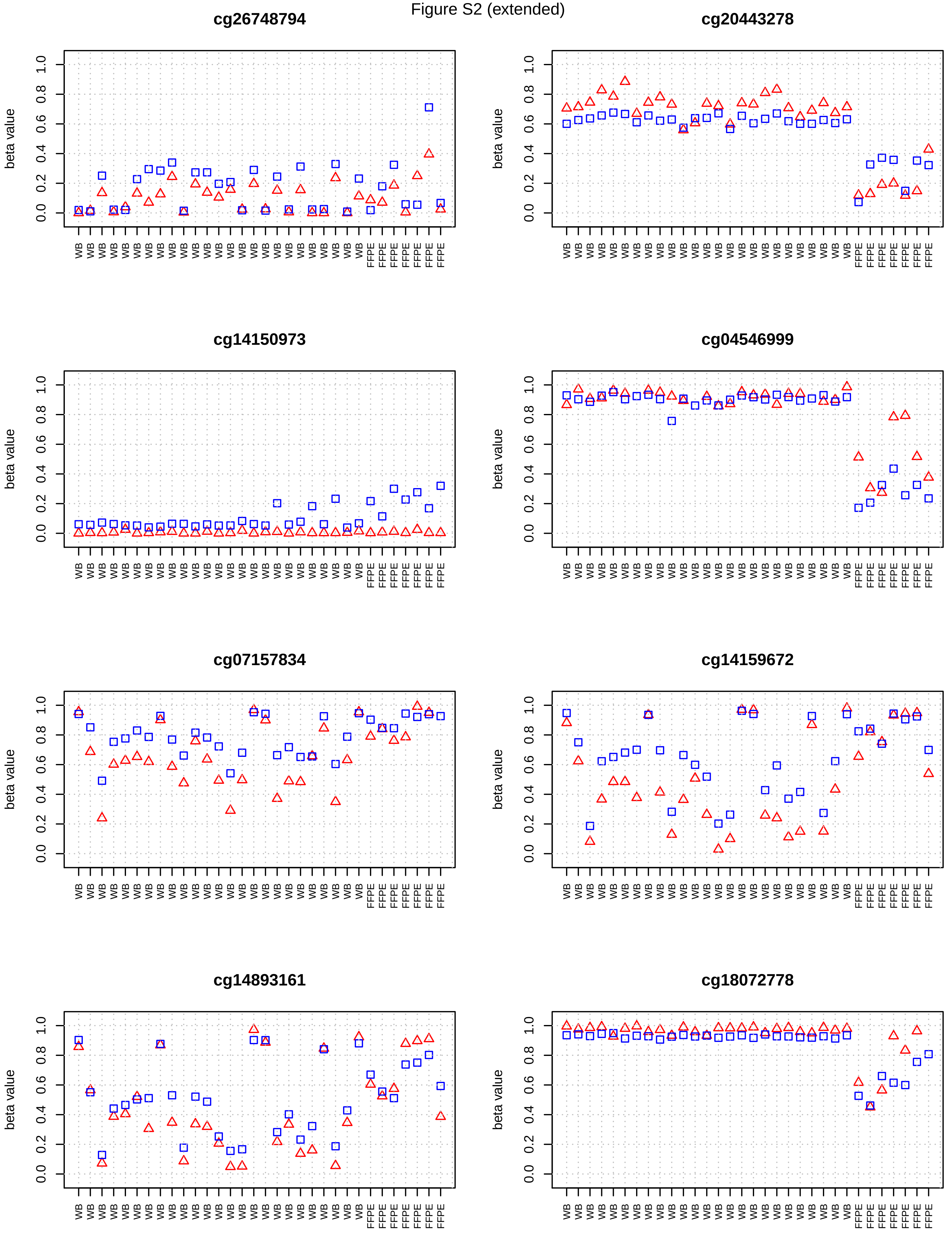

Figure S2 (extended)

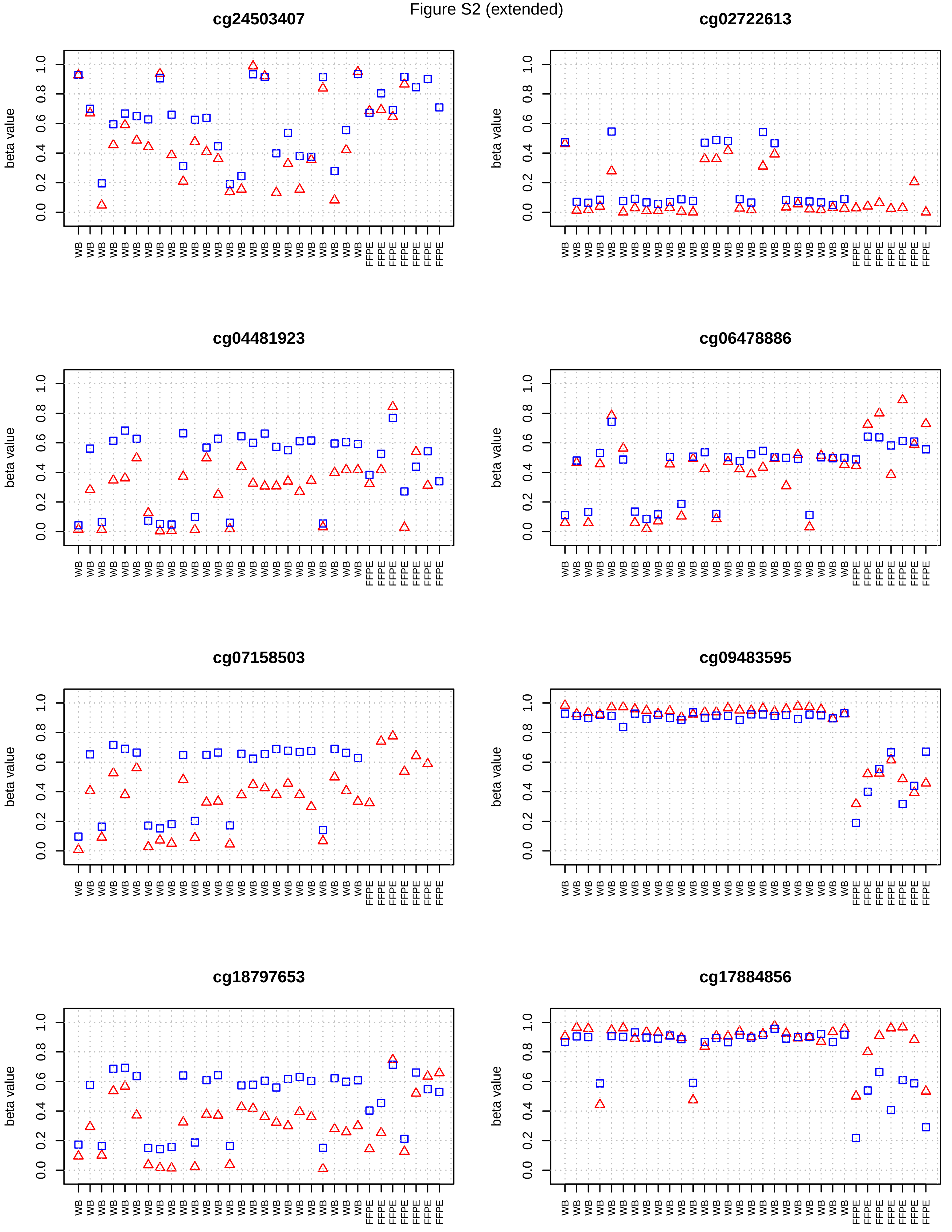

Figure S3

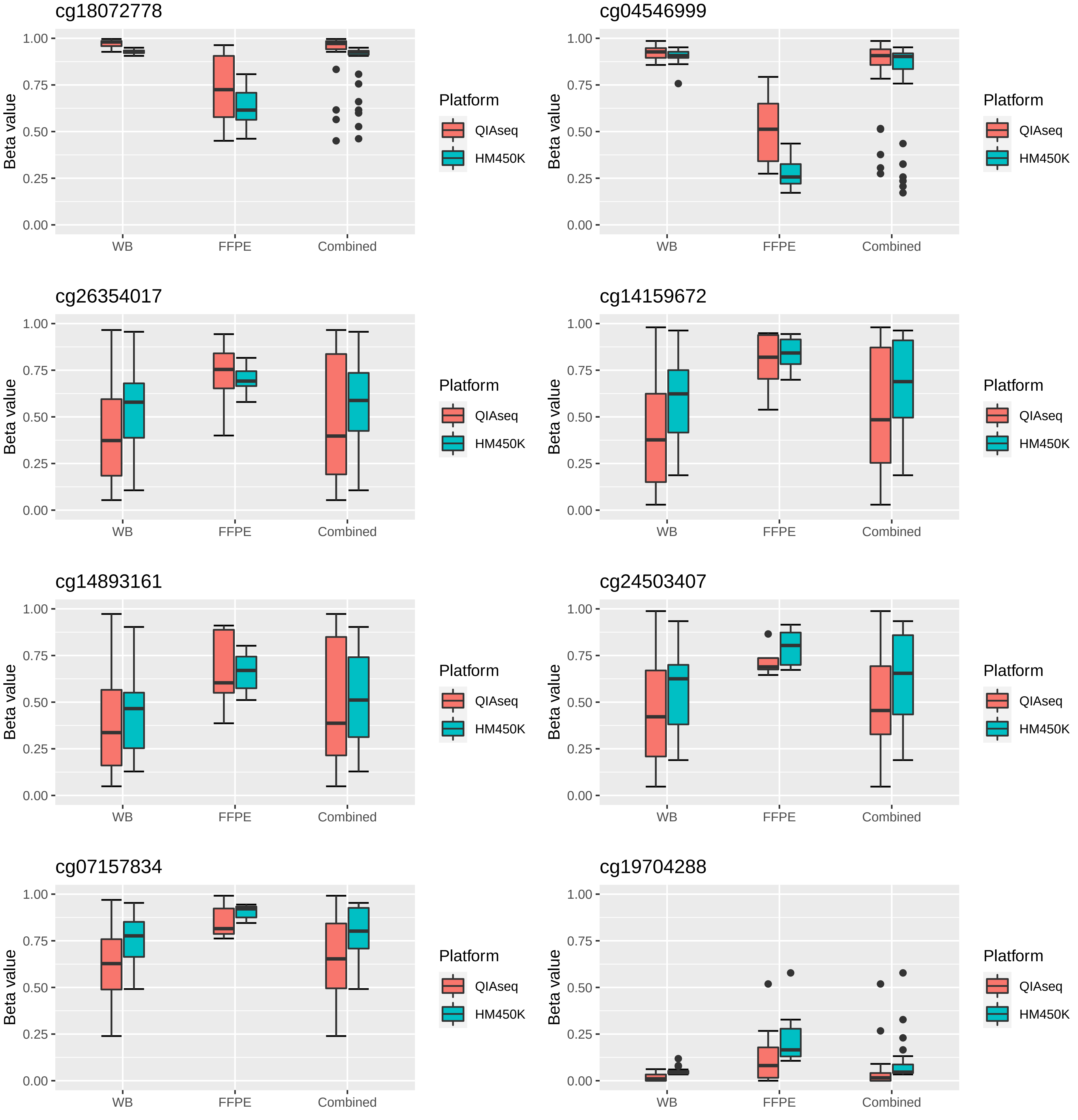

cg18072778
cg04546999
cg26354017
cg14159672
cg14893161
cg24503407
cg07157834
cg19704288
Beta value
1.00
0.75
0.50
0.25
0.00
WB
FFPE
Combined
Platform
QIAseq
HM450K

Figure S3 (extended)

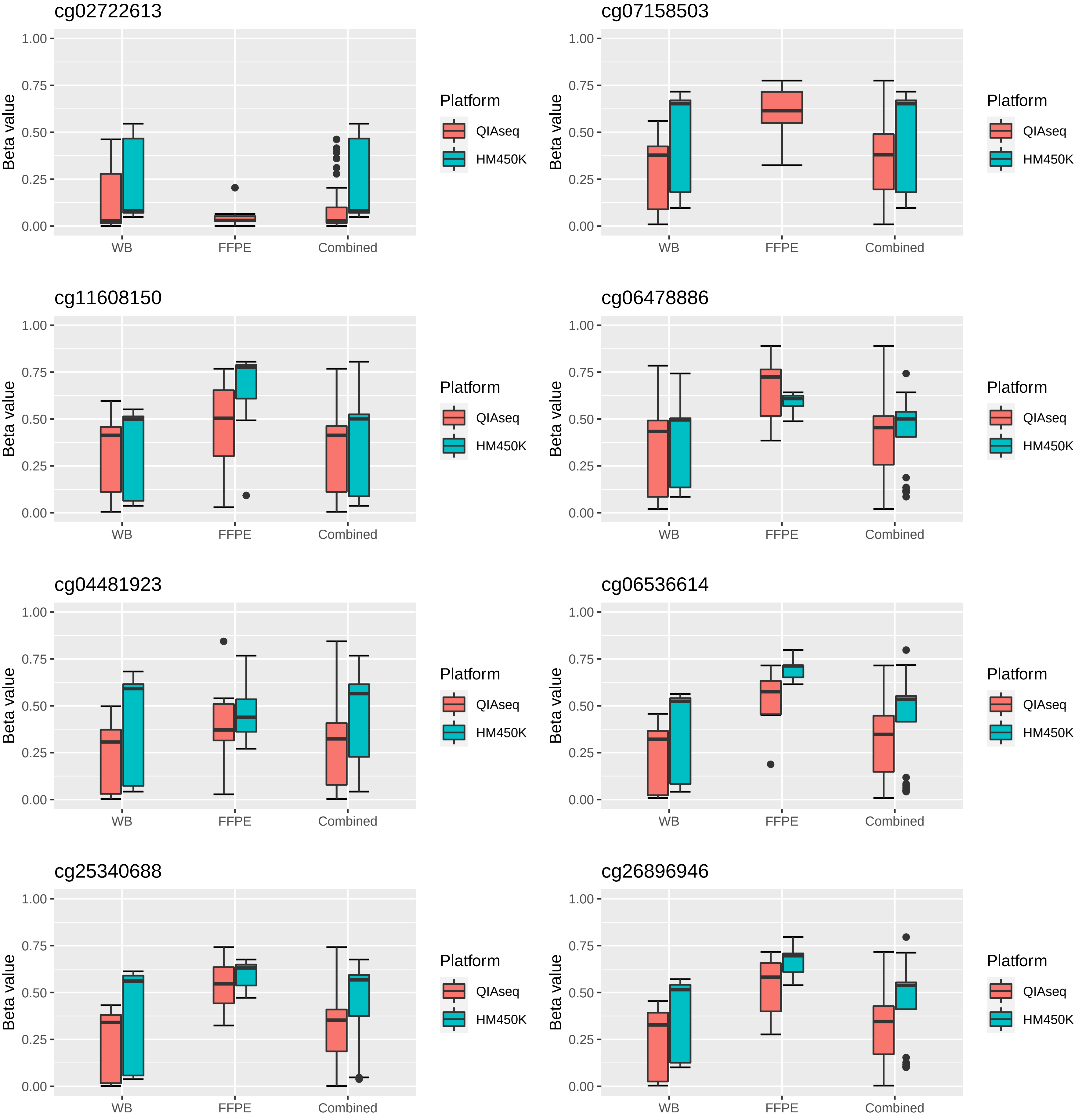

Figure S3 (extended)

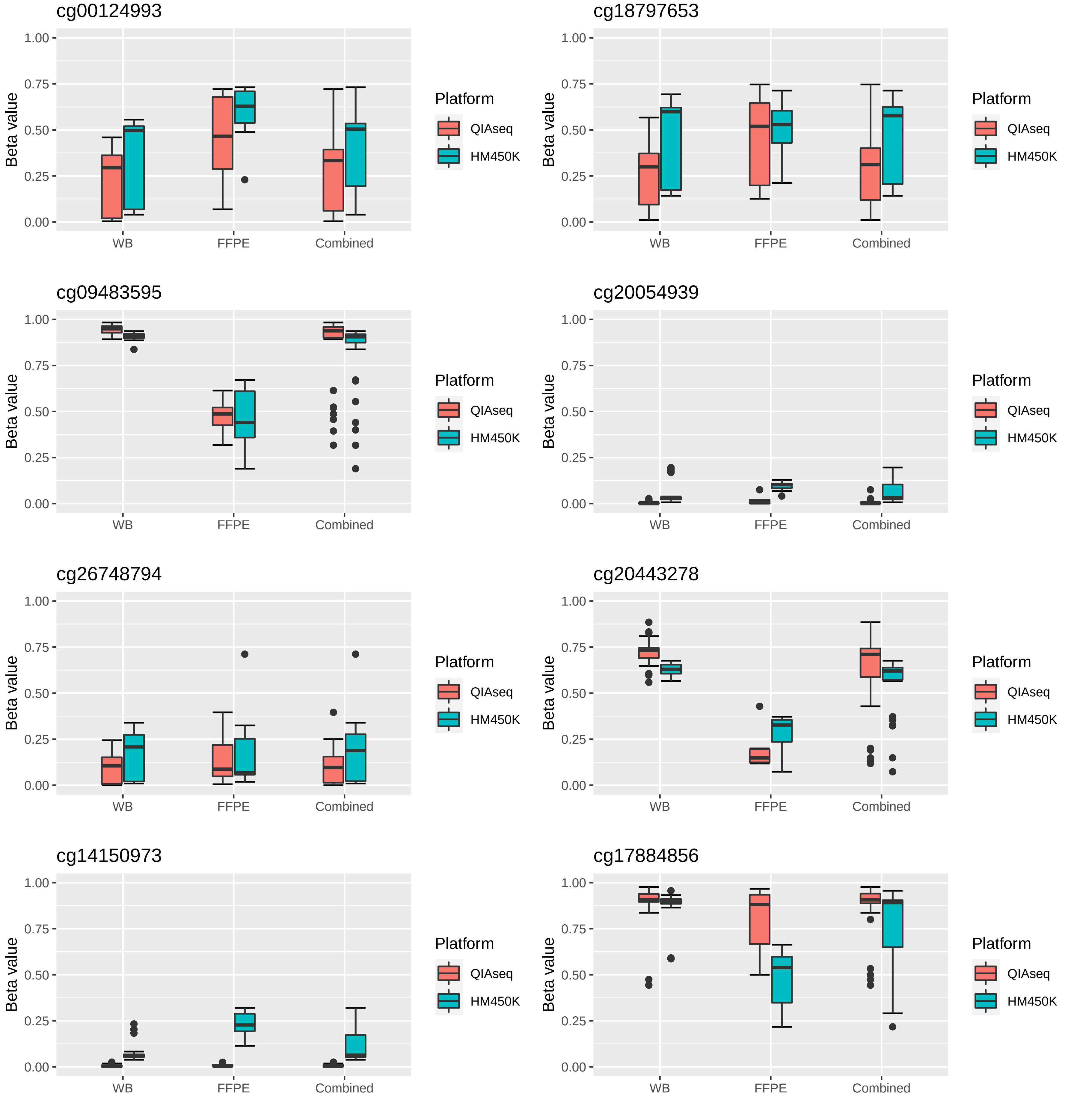

Figure S4

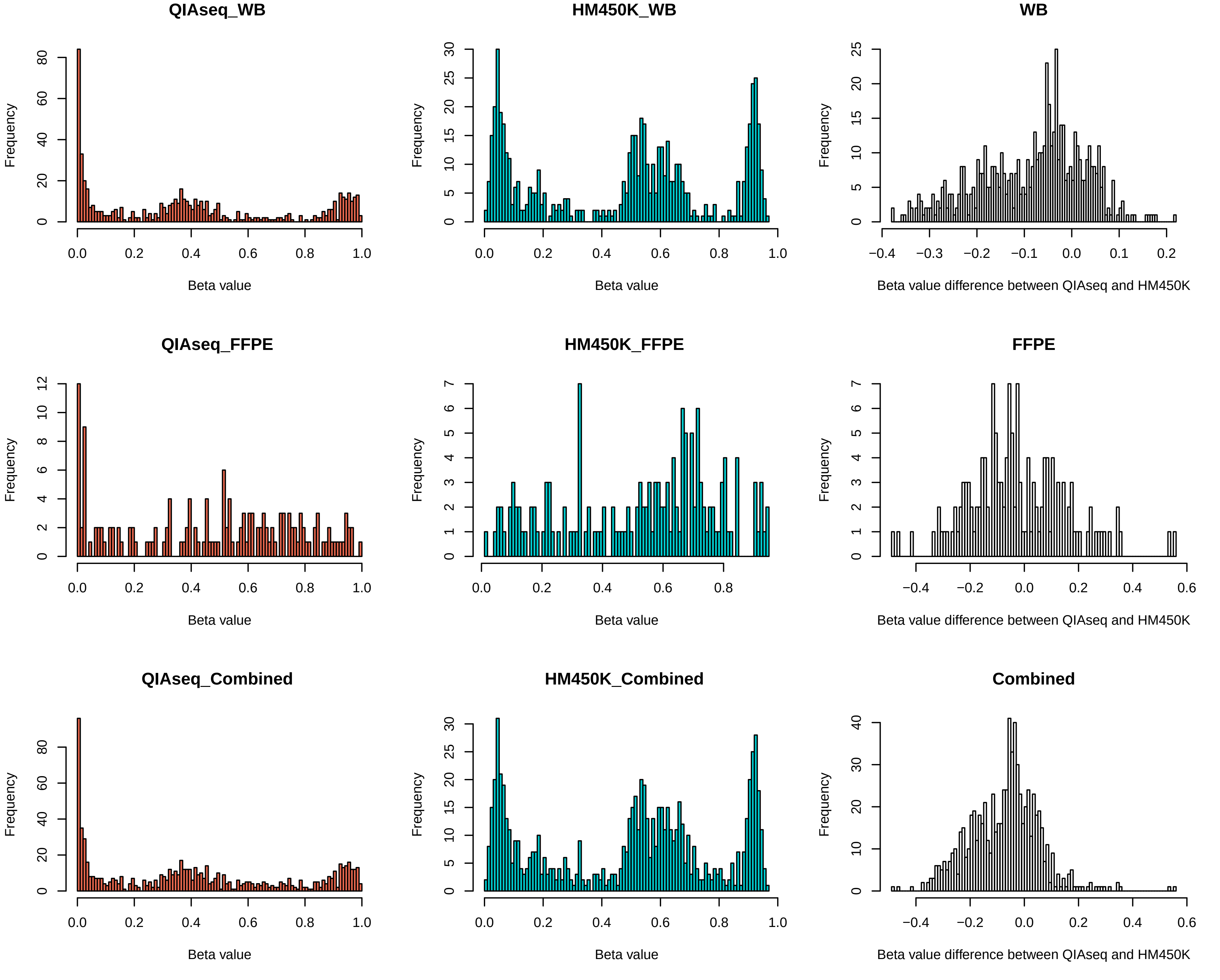

QIAseq_WB
HM450K_WB
WB
QIAseq_FFPE
HM450K_FFPE
FFPE
QIAseq_Combined
HM450K_Combined
Combined
Frequency
Beta value
Beta value difference between QIAseq and HM450K

Figure S5

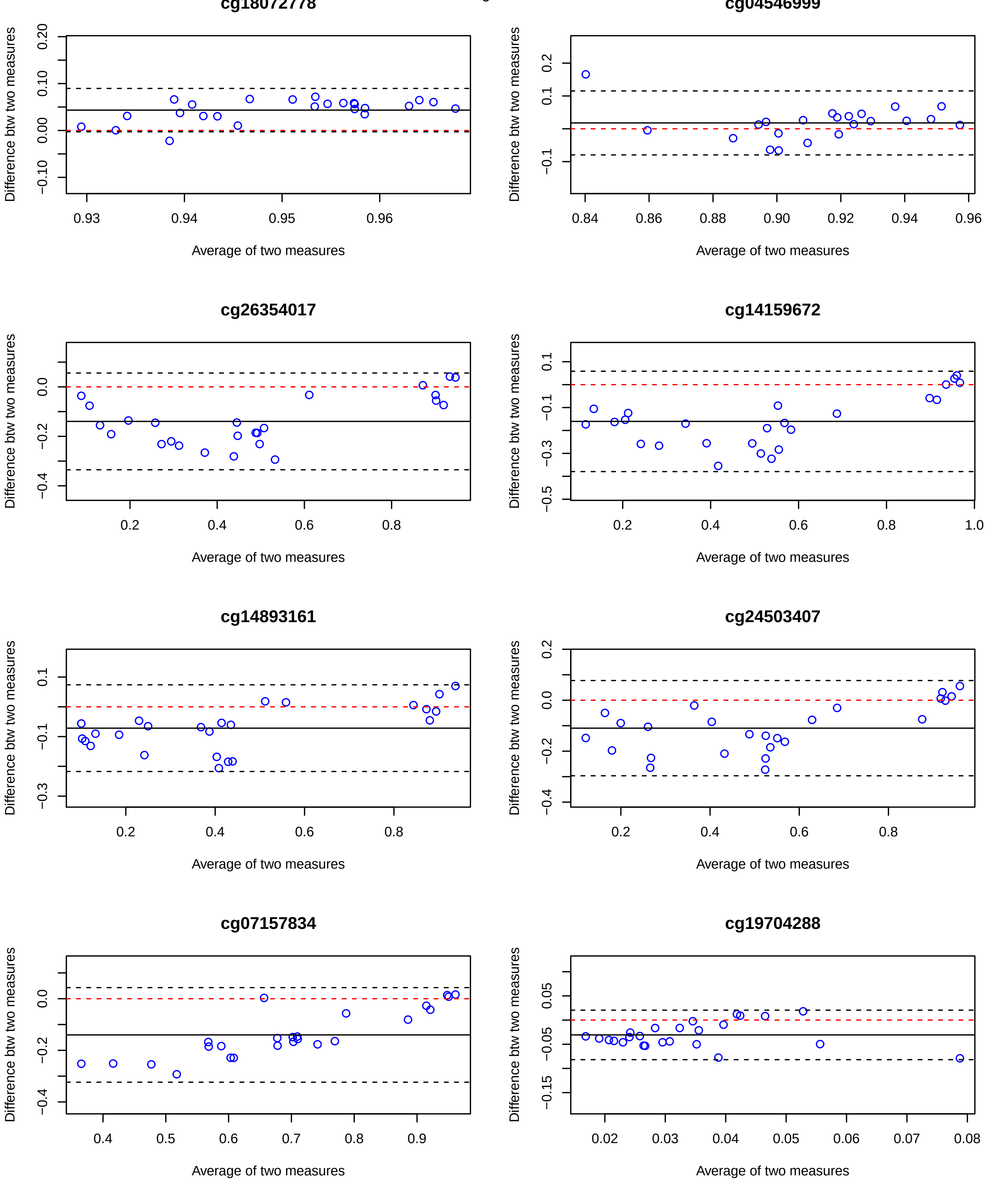
cg18072778
cg04546999
cg26354017
cg14159672
cg14893161
cg24503407
cg07157834
cg19704288
Difference btw two measures
Average of two measures
−0.10 0.00 0.10 0.20
0.93 0.94 0.95 0.96
−0.1 0.1 0.2
0.84 0.86 0.88 0.90 0.92 0.94 0.96
−0.4 −0.2 0.0
0.2 0.4 0.6 0.8
−0.5 −0.3 −0.1 0.1
0.2 0.4 0.6 0.8 1.0
−0.3 −0.1 0.1
0.2 0.4 0.6 0.8
−0.4 −0.2 0.0 0.2
0.2 0.4 0.6 0.8
−0.4 −0.2 0.0
0.4 0.5 0.6 0.7 0.8 0.9
−0.15 −0.05 0.05
0.02 0.03 0.04 0.05 0.06 0.07 0.08

Figure S5 (extended)

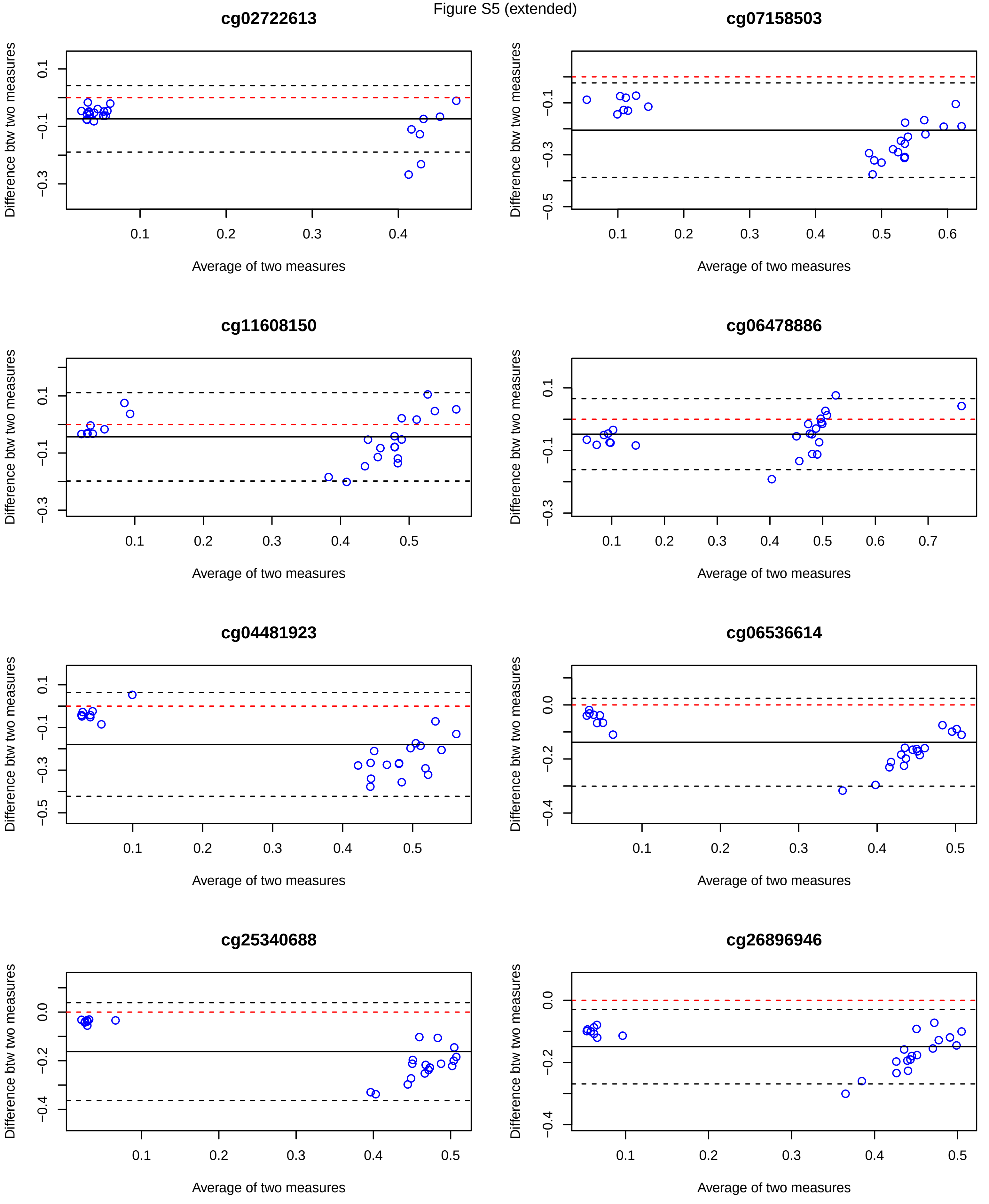

Figure S5 (extended)

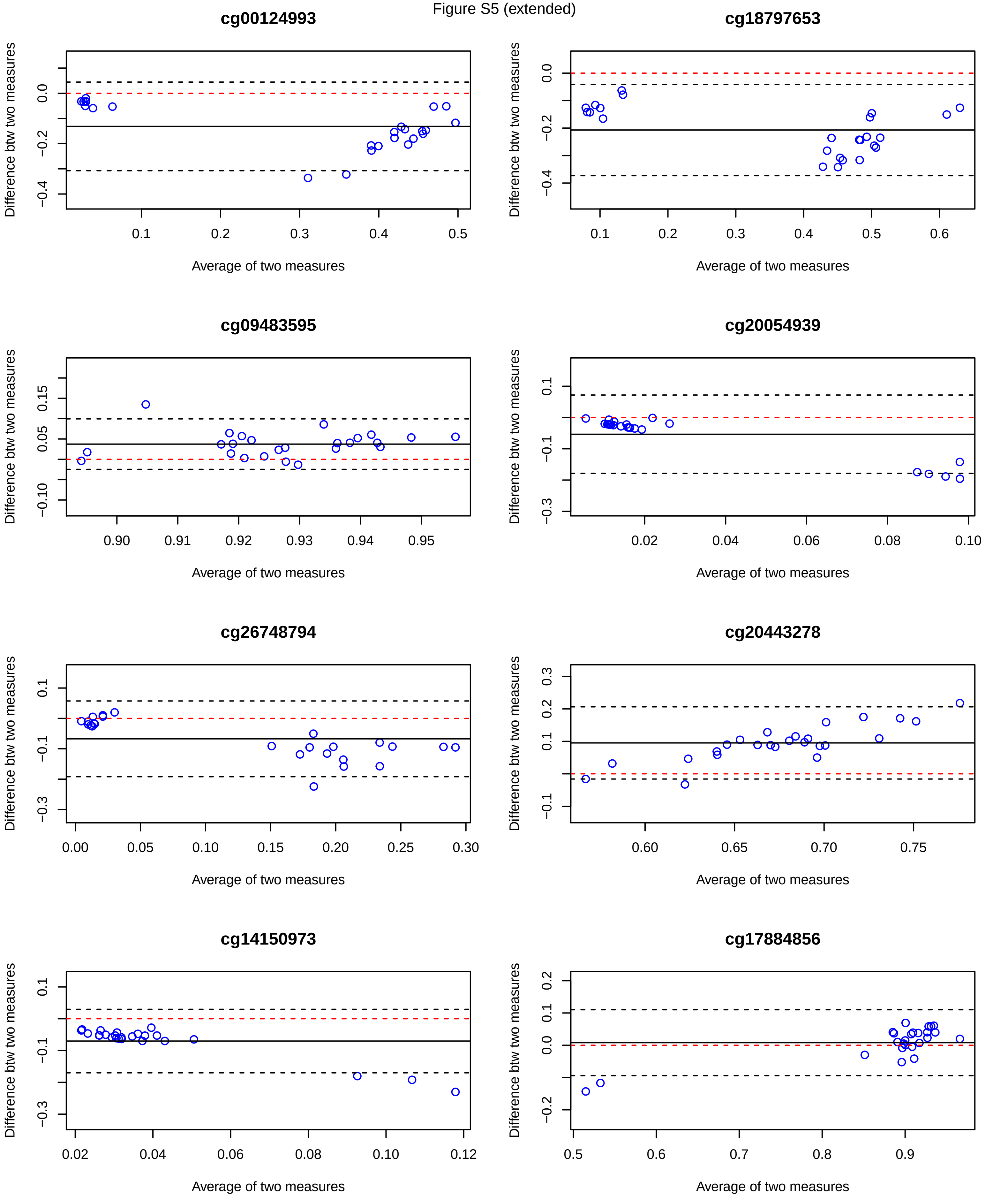

Figure S6

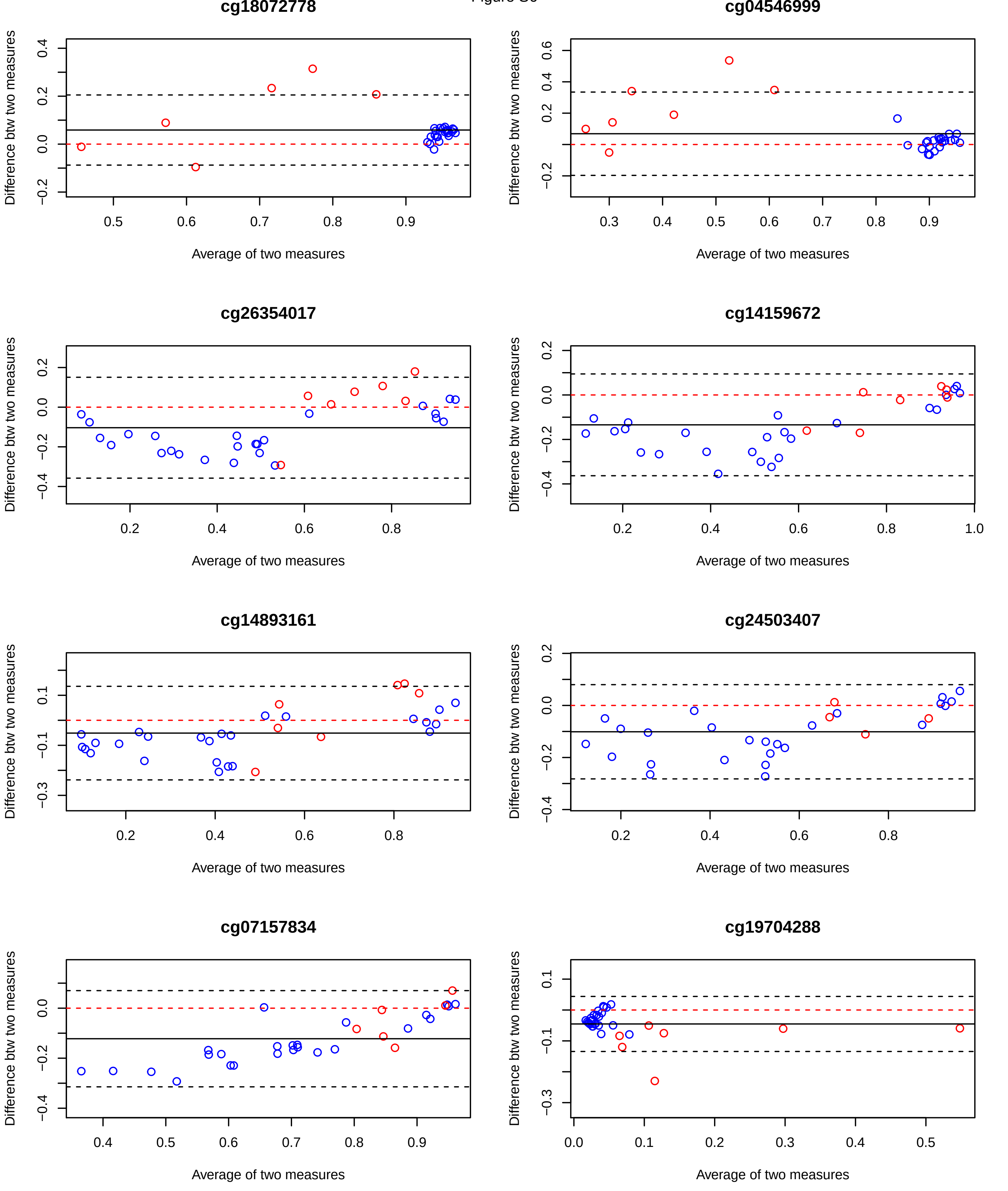

cg18072778
cg04546999
cg26354017
cg14159672
cg14893161
cg24503407
cg07157834
cg19704288
Difference btw two measures
Average of two measures

Figure S6 (extend)

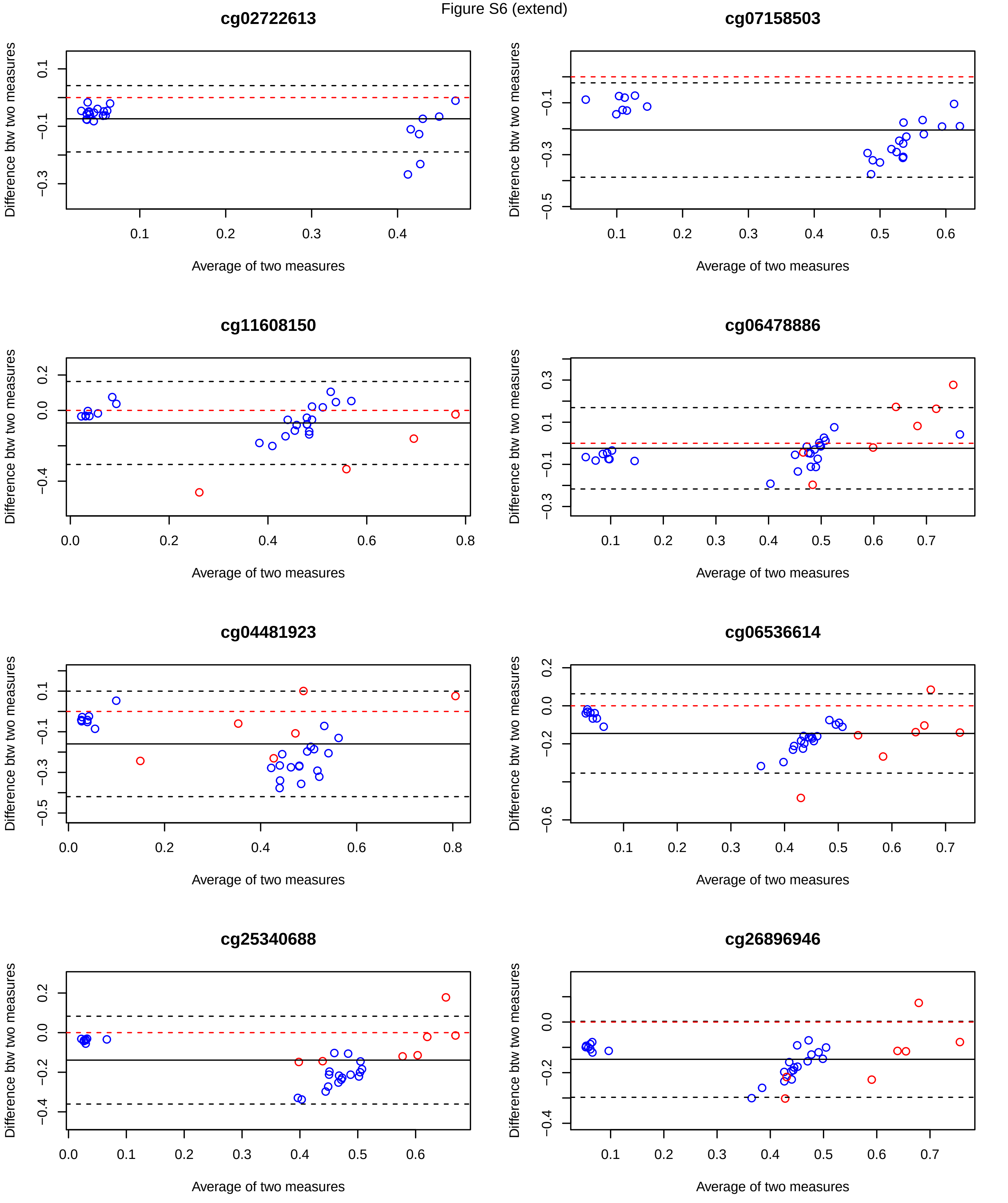

Figure S6 (extend)

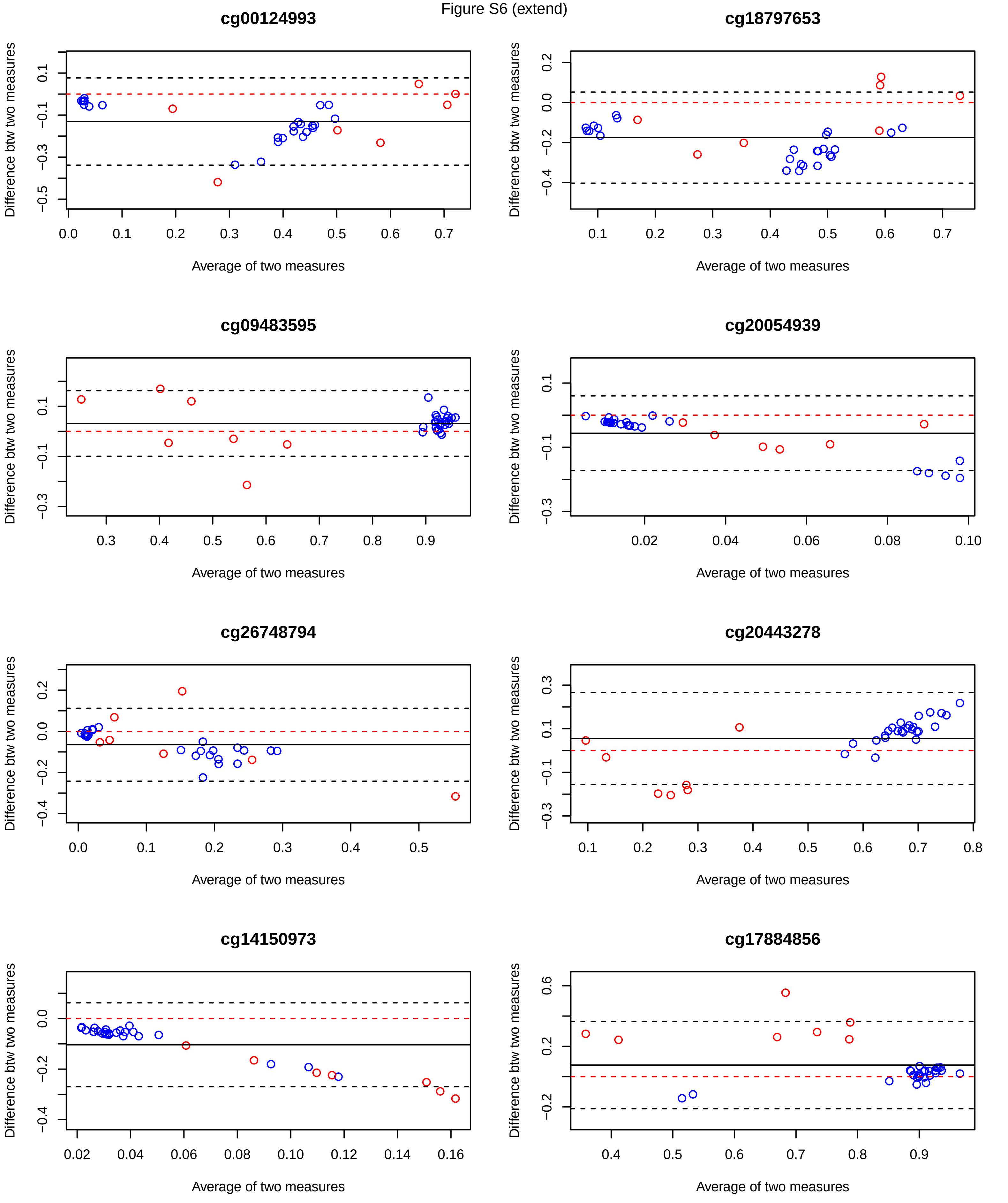

Supplement: Supplementary file 1 — Additional file 1: Supplementary Tables and Figures. [file 13104_2021_5809_MOESM1_ESM.pdf]
